# Using a Theoretical Framework to Investigate Whether the HIV/AIDS Information Needs of the AfroAIDSinfo Web Portal Members Are Met: A South African eHealth Study

**DOI:** 10.3390/ijerph110403570

**Published:** 2014-03-28

**Authors:** Hendra Van Zyl, Marike Kotze, Ria Laubscher

**Affiliations:** 1 Web and Media Technologies, South African Medical Research Council, P.O. Box 19070, Tygerberg 7505, South Africa; E-Mail: mmyburgh@gmail.com; 2 Biostatistics Unit, South African Medical Research Council, P.O. Box 19070, Tygerberg 7505, South Africa; E-Mail: ria.laubscher@mrc.ac.za

**Keywords:** consumer health informatics, eHealth, HIV/AIDS, theoretical framework, health information needs

## Abstract

eHealth has been identified as a useful approach to disseminate HIV/AIDS information. Together with Consumer Health Informatics (CHI), the Web-to-Public Knowledge Transfer Model (WPKTM) has been applied as a theoretical framework to identify consumer needs for AfroAIDSinfo, a South African Web portal. As part of the CHI practice, regular eSurveys are conducted to determine whether these needs are changing and are continually being met. eSurveys show high rates of satisfaction with the content as well as the modes of delivery. The nature of information is thought of as reliable to reuse; both for education and for referencing of information. Using CHI and the WPKTM as a theoretical framework, it ensures that needs of consumers are being met and that they find the tailored methods of presenting the information agreeable. Combining ICTs and theories in eHealth interventions, this approach can be expanded to deliver information in other sectors of public health.

## 1. Introduction

### 1.1. Background

The HIV/AIDS epidemic has led to the most serious present-day global challenge for public health. Consequently, unprecedented action began in 2000 with the initiation of the 6th United Nations Millennium Development Goal aiming to halt and reverse the AIDS epidemic by 2015. The current status according to the 2013 Global Report of the Joint United Nations Programme on HIV/AIDS (UNAIDS) [1], indicates that there are 35.3 million people living with HIV and AIDS worldwide. The worst affected region is sub-Saharan Africa where 70% of new HIV infections occurred during 2012. In their review of South African health challenges, Mayosi *et al.* [2] emphasized the urgent need to increase HIV prevention efforts. They identified insufficient HIV knowledge and risk perceptions, as posing major stumbling blocks to HIV/AIDS prevention in South Africa. While seeking for innovative approaches, Information and Communication Technologies (ICTs) in low and middle income countries (LMICs) were considered appropriate and showed potential to provide HIV/AIDS preventative education [3,4,5]. eHealth, an emerging field at the intersection of health informatics and public health, was identified by Eysenbach [6] as a suitable method of delivering health services and information through the use of ICTs. Furthermore, he described eHealth as not merely a technological development but a state-of-mind through which, for example, benefactors of eHealth can become empowered in a patient-centered approach to make informed health choices. Factors influencing uptake of eHealth solutions include usefulness and comprehension, ease of use, reliability of content, as well as accessibility and acceptability to ICTs, while these are directly related to collectively meeting information needs of potential users. 

In the South African context, few studies have been undertaken where these factors have been investigated. The South African Medical Research Council (MRC) showed how tailoring information according to the target groups’ information needs and preferences in an eHealth intervention, better recall and understanding of HIV/AIDS knowledge was achieved by pupils in an eLearning Web-based platform compared to classroom instruction [4]. In contrast, another South African eHealth study qualitatively explored the factors that would influence ICT acceptability in the form of telemedicine solutions in healthcare settings in the rural Eastern Cape Province [5]. Outcomes indicated that structural barriers such as insufficient infrastructure, a lack of eSkills and quality health information posed the greatest barriers to uptake of ICT-based solutions.

As opposed to the use of ICTs, how knowledge was shared and communicated by healthcare providers was qualitatively examined in a South African/Ugandan study on HIV/AIDS/cancer information needs of patients and caregivers [7]. Findings uncovered the negative outcomes when insufficient knowledge on the diseases and insensitive communication by healthcare providers, rendered patients unable to manage their diseases, hereby increasing social challenges on support and finances. This further highlights the crucial link between language and understanding in order for consumers to make informed health decisions, which led to an attempt in South Africa for the development of medical terms in indigenous languages [8].

The above suggests that public health practitioners need more than ICTs and health information for eHealth interventions to be effective. This paper sets out to investigate if a theoretical framework can ensure that information needs of patients are addressed comprehensively and according to their preferences.

### 1.2. Theoretical Framework

The field of Consumer Health Informatics (CHI) has emerged to level the relationship between health professionals and lay people by benefitting health professionals and assisting patients and community members as “health consumers” to make informed choices regarding their health [9]. CHI evolved from health informatics, incorporating public health, health education and promotion, as well as library and communication sciences. Key areas include (a) analysis of health consumers’ information needs, (b) researching and implementing techniques for accessibility of health information, (c) developing approaches to integrate consumer preferences in information systems, and (d) researching impact of CHI outcomes on public health. In this way, it empowers health consumers to improve health through tailored health information which is often linked to health education. The Internet, although not exclusive, is an important conduit through which consumers can access health information but raises the question of quality and reliability which is often left to be judged by consumers.

Linked to the CHI theoretical framework, a psychosocial approach, the Web-to-Public Knowledge Transfer Model (WPKTM), is an eHealth technique to guide the development of health Web sites [10]. Consisting of two phases, the first phase of the WPKTM includes planning and consists of generic principles guiding identification of the targeted consumers’ information needs. It addresses the need to identify an audience, establish what information would be relevant and required, classification of health information according to health literacy levels, and highlighting relevance of new knowledge while avoiding information overload and encouraging utilization. During the second, or implementation phase, information needs of consumers will be converted into technology which involves a range of good practice guidelines to develop a health Web site [10]. Included is emphasis on implementation of quality assurance methods to remove the responsibility from health consumers of judging content for reliability. This incorporates an editorial board to review articles, articles written by professional science writers, referencing of articles and Web site certification e.g., by the Health on the Net Foundation. Their HONcode is based on a set of ethical principles and certification implies that if the review indicates the health Web site is in compliance with the HONcode, its seal is displayed on the Web site showing visitors that it is a reputable health information repository [11].

### 1.3. The Present Study

Mindful of public health challenges to respond effectively to HIV/AIDS prevention in South Africa and to provide appropriate health information to diverse groups, the MRC has developed a Web portal on HIV/AIDS. The theoretical framework presented by CHI and the WPKTM guided identification of the consumer groups and their information needs, followed by converting of their needs into an AIDS information portal, AfroAIDSinfo ^TM^ [12]. The consumer groups included the public, health professionals, policy makers, educators and scientists. As suggested in the key areas of CHI, regular eSurveys are conducted to investigate if AfroAIDSinfo continued to meet the information needs of its five consumer groups. This was considered essential due to continuous developments in the field of HIV/AIDS, implying that knowledge on the disease should be up to date, translated and tailored according to respective groups’ preferences and packaged according to complexity levels to aid in utilization [13]. For instance, assisting patients to make informed health choices, guide policy makers on decision-making, or informing health professionals on appropriate practices. 

The objectives of the study included the need to identify consumers’ demography in order to investigate if the five consumer groups’ information needs were comprehensively addressed and information considered useful. This research is aimed at contributing to the small pool of current knowledge on effective use of ICTs in eHealth interventions for HIV/AIDS prevention across diverse consumer groups. 

## 2. Material and Methods

### 2.1. Design and Sampling

Epistemologically, a positivist approach was followed based on a cross-sectional study design [14] and which took place between January and December 2013. Since visitors to the AfroAIDSinfo Web portal are invited to register as members, the sampling frame consisted of the AfroAIDSinfo subscribed membership database. Sampling was conducted as a census as all members were eligible and invited through a special eNewsletter to participate in the eSurvey. The questionnaire underwent testing and evaluation in previous studies and has been found to have established face and content validity. Reliability was established through piloting the questionnaire. An institutional review board also reviewed the questionnaire during two previous CHI studies. Response bias was controlled for by pre-testing the questionnaire to ensure that survey questions were clear and that where categories such as age groups were used, that these did not overlap. Ethics approval for the research was given by the MRC Ethics Committee which included scientific review. The eNewsletter through which members were invited to participate in the study, included an explanation of the research as well as a link to the eSurvey on the AfroAIDSinfo Web portal. The research instrument was an online questionnaire but before accessing it, a Participant Information Sheet including an explanation of ethical issues, such as voluntary participation, freedom to withdraw from completing the online questionnaire, confidentiality of collected data and anonymity of participants was mandatory reading. Participants had to consent for participation by clicking a button before the online questionnaire was displayed.

### 2.2. Data Collection and Analysis

The majority of questions were close-ended, starting with a section for demographic data collection in particular the consumer group in which a participant was registered on AfroAIDSinfo. The second section consisted of questions on meeting consumer groups’ information needs.

The data was captured in Excel spreadsheets and converted to SAS version 9.2 (SAS Institute Inc., Cary, NC, USA) for analyses. Frequencies were calculated on all the variables and the Chi-square test with a *p* < 0.05 indicated statistical significance. The consumer group variable was used to assess the association between the different groups and multiple aspects contributing to meeting consumer groups’ information needs. A composite score was calculated on a set of binary variables to assess how well information needs of individual consumer groups were met. These included self-assessment as to whether information needs were met; reliability of content; using the content; reading the monthly articles; usefulness of the monthly eNewsletters; and formatting of the eNewsletter according to preferences.

Thereafter, an average score of the different consumer groups was compared by fitting a logistic regression model with the largest group, Health Profession, as the reference group. Results between the present 2013 and the previous 2010 eSurveys were compared to assess whether the consumer groups’ profiles have changed. 

Three open-ended questions were included to explore critical questions such as why information was perceived as reliable; if articles were considered useful, how this information was used; and opportunities to make suggestions for topics not currently addressed in articles and eNewsletters. These were qualitatively analysed using Thematic Content Analysis [15] as this is an inductive method to rigorously categorize segments of qualitative data into meaningful themes which contributes to interpretation of textual data. 

Three invitations to participate in the eSurvey were distributed to the AfroAIDSinfo membership database from January to March 2013. 

## 3. Results

At the time of the eSurvey, there were 1,824 members in the AfroAIDSinfo subscription database and of these, 83 members completed the online questionnaire, a response rate of 4.6%. In relation to the complete population of AfroAIDSinfo consumer groups, respondents’ representativeness for Science was 5.5% (n = 15 of 275); 4.9% (n = 15 of 305) for Public; 2.7% (n = 5 of 184) for Policy; 4.8% (n = 24 of 500) for Health Profession; and 4.3% (n = 24 of 560) for Education. Results below include the demographic profile of participants, the extent to which information needs were met, including usefulness of information, and a comparison of results in this study to the 2010 eSurvey in order to monitor the efficacy of the theoretical framework over time.

### 3.1. Health-Seeking Behavior

#### 3.1.1. Demographic Profile

**Figure 1:**
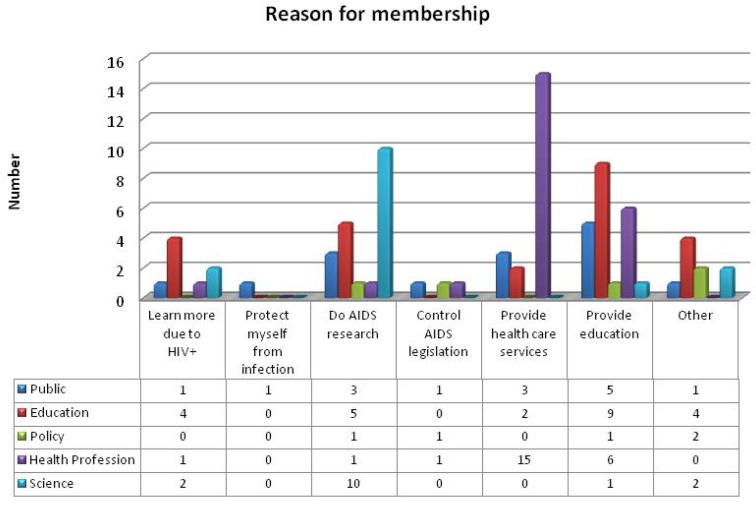
Reasons why respondents’ became members of AfroAIDSinfo.

The demographics of respondents in Table 1 not only provide insights into their overall profile but also demonstrated their health-seeking behavior. In addition to the demographic profile, Figure 1 below shows what motivated respondents to become AfroAIDSinfo members. Unexpectedly, the Education consumer group had the highest number of HIV positive respondents. Overall, only one respondent became a member to gain HIV/AIDS information for personal protection who, as might be expected was a member in the Public consumer group. In the *Other* category, several policy-related reasons were given, for personal studies and a deviant response hinting that membership was to track whether HIV is a man-made virus and the cure withheld from Africans.

**Table 1:** Demographic profile of respondents (n = 83).

Characteristics	%	Explanation
Gender	35% (n = 29)	Males
	65% (n = 54)	Females
Consumer group	29% (n = 24)	Health Profession and Education consumer groups had the highest respondent rates; *Public and Science: 18% (n = 15) each; Policy:6% (n = 5)*
Age	53.1% (n = 44)	Highest response rates were among ages 35-54 years; *Age group 55 and above: 26.5% (n = 22); Age group 15-34 years: 20.5% (n = 17)*
Qualifications	53% (n = 44)	Highest response rates were among post-graduates; *Degree/diploma/certificate: 37.3% (n = 31); Senior school: 8.4% (n = 7); Junior school: 1.2% (n = 1)*
Country	74% (n = 62)	South Africa; *In total, 82% (n = 68) were from 5 African countries and 18% (n = 15) from the USA and European countries*
Length of membership	37% (n = 31)	Highest response rate was among those with 1–3 years membership; *4–6 years: 31.3% (n = 26); <1 year: 19.3% (n = 16); 7–9 years: 12% (n = 10)*
Profession related to consumer group	87% (n = 72)	Confirmed that they registered in a consumer group related to their professions

#### 3.1.2. Relevance of Information Influencing Health-Seeking Behavior

Health-seeking behavior demonstrated three peaks as to when the AfroAIDSinfo Web portal was most often visited. The peaks were influenced by how relevant respondents found information for a given period. The highest frequency of visits were when consumers needed HIV/AIDS information (43.17%, n = 32), revealing that they regarded AfroAIDSinfo as a preferred HIV/AIDS repository. This was followed by weekly visits (25.83%, n = 23) when current awareness news feeds were updated and monthly visits (17.33%, n = 44) when new articles appeared for each consumer group and the eNewsletter was distributed.

The fact that the majority of respondents’ occupations were related to the consumer groups in which they had registered, contributed to the finding that the AfroAIDSinfo Web portal provided relevant HIV/AIDS information according to the expectations of its membership. Notably, government employees were spread across all the consumer groups, and not only in the Policy group.

In the Public consumer group relevance of information on various aspects of HIV/AIDS was shown to be the main reason for membership, followed by the gaining of information on the latest research and the strengthening of health care services. The Public group included community members, patients, caregivers, NGOs, administrators, health service providers and government representatives. In all other consumer groups, relevance of information influencing health-seeking behavior was shown according to the focus of the group. As guided by the WPKTM, the approach used to improve understanding was to develop separate information for each consumer group [12]. Furthermore, each consumer group’s information was tailored relevant to their information needs and packaged according to levels of complexity thus preventing information overload and leading to increased utilization.

The latest articles for each consumer group are accessible from the AfroAIDSinfo home page while the complete repository requires logging in. To establish the depth of their health-seeking behavior respondents were asked as to whether they had logged into the AfroAIDSinfo Web portal to read previous articles. The Public consumer group seemed to be the most willing to log in to find additional information, rated at 28%. They were followed by the Health Profession (24%), Education (21%), Science (19%) and Policy (8%) groups. 

### 3.2. Meeting Information Needs

In both the Public and Policy consumer groups, respondents rated all their information needs as being met (100%). The rating in Education was 96% (n = 23), followed by 93% (n = 14) in Science and 88% in Health Profession.

#### 3.2.1. Reliability of Information

In keeping with the high rating as referred to above, an important characteristic of the AfroAIDSinfo Web portal were the various quality assurance methods implemented to ensure credibility and reliability of the HIV/AIDS information. Three consumer groups, Public, Policy and Health Profession rated reliability of content at 100%, followed by 93% (n = 22) by Education and 88% (n = 13) by the Science consumer groups. Due to the importance of reliability, an open-ended question allowed respondents to motivate their ratings and these were qualitatively analyzed. Five themes emerged which collectively supported quantitative results. These included *Educative usefulness* (29%) because information was perceived as credible and therefore useful to train others and fill knowledge gaps. *Updated with latest research* (25%) was strongly linked to reliability due to regular provision of the latest research findings, making AfroAIDSinfo a preferred knowledge resource. Respondents showed a high regard for the *MRC branding* (23%) due to the professionalism with which these articles are written, reaffirming the MRC reputation as a science council which produces high quality research. Information was packaged according to the complexities of the different groups’ levels, focusing on developments in Africa, and combining reliability to making it *Relevant to needs* (12%). Finally, *Quality assurance* (11%) was perceived as self-evident due to editorial reviews, references enabling verification and AfroAIDSinfo carrying HONcode certification to strengthen overall trustworthiness.

#### 3.2.2. Usefulness of Articles and eNewsletters

Related to reliability, was the extent to which respondents felt confident to use information. The usefulness of the articles and especially the eNewsletters was highly rated in all the consumer groups (Table 2).

**Table 2:** Respondents reading monthly articles and eNewsletters.

Consumer groups	Monthly articles	Monthly eNewsletter
Health Profession	100% (n = 24)	96% (n = 23)
Public	93% (n = 14)	100% (n = 15)
Science	93% (n = 14)	93% (n = 14)
Policy	80% (n = 4)	100% (n = 5)
Education	79% (n = 19)	100% (23)

The extent as to how the provided information was used, was explored in an open-ended question. Five themes emerged after qualitative analysis. The *Education* (42%, n = 25) theme emerged as the most important activity and was used not only to fill own knowledge gaps as well as for community education, creating awareness in school settings, university lecturing and HIV/AIDS peer education. The next theme, *Referencing and research* (24%, n = 14) showed that information was used as a source of reference, made easy by citations provided with each online article, and referred to in conference presentations, graduate studies, own research and articles. *Dissemination* (15%, n = 9) emerged as another theme of reliability where the AfroAIDSinfo resources were distributed in departmental emails, on social media, in own newsletters and among teachers, pupils and patients. A theme, *Own interest* (12%, n = 7), included personal empowerment for health decision-making and improving own knowledge, together with mentoring of staff in primary health care facilities, personal studies and making contact with others working in similar areas of HIV/AIDS. The final theme, *Policy purposes* (7%, n = 4) highlighted government representatives who used the information for interaction with ministries in other sub-Saharan African countries, writing of policy documents, reports, informing legislature, policy decision-making and policy-related public speeches.

Respondents could suggest topics for articles to further support usefulness of the AfroAIDSinfo Web portal content. It was previously found that this opportunity contributed to building a relationship with members as new articles written on suggested topics were indicated in the eNewsletter. 23% (n = 19) of respondents proffered suggestions while 77% (n = 64) indicated that they were satisfied that their information needs were being met. A similar question was asked about the eNewsletter content with 72% (n = 60) of respondents satisfied that their information needs were being met while 27% (n = 22) made further suggestions.

#### 3.2.3. Information Meeting User Preferences

Since the previous eSurvey in 2010, modifications were made to improve articles according to user preferences which also contributed to the usefulness and ease of reusing information. A summary was added to the beginning of each online article giving a brief overview of the article. This addition was rated by respondents at 100% according to their preferences. At the end of each article a suggested citation was added to improve ease to cite the article. 42% (n = 35) of respondents indicated having used these citations. Overall usefulness of these additions were rated at 95% (n = 79) by respondents.

Due to the importance of the eNewsletter as a push technology to disseminate customized HIV/AIDS information to the various consumer groups, respondents was asked to rate the eNewsletter format. Overall, acceptability of the current format, layout and content, was rated at 95% (n = 79). In the Public (n = 15) and Policy (n = 5) consumer groups, respondents were 100% satisfied with the format, 96% (n = 23) in Health Profession, 93% (n = 14) of respondents in Science and 91% (n = 22) in Education. An open-ended question qualitatively explored if respondents would like to make further suggestions to address their preferences. Only a few responses were made to reflect the general respondent satisfaction. These included making the eNewsletter available in pdf format thus not solely embedded in emails and to enhance it with the use of more graphics. Sometimes, news items were included from external Web sites that required registration. Some respondents saw this as a hindrance. The length of the eNewsletter was overall rated as just right (93%, n = 77) with 3.6% (n = 3) finding it either too long or alternatively too short.

Social networking presences in both Twitter and Facebook contributed in informing visitors of newly published articles, disseminating new developments in HIV/AIDS and as an interactive platform. Awareness of these social media presences when assessed, showed that 44% (n = 37) of all respondents were aware of these presences with the highest visits from the Policy consumer group (80%), followed by the Public group at 60%. 

### 3.3. Information Needs Met in 2013 Compared to 2010

In order to monitor trends according to which information needs were met over time, a comparison was made of the results of this 2013 study and those of the 2010 eSurvey. Table 3 shows the univariate analysis results for both 2010 and 2013, categorized by consumer group. Six criteria collectively contributed to the meeting of information needs. Criteria included how competently information needs were being met; reliability of content; using of the content; reading of the monthly articles; usefulness of the monthly eNewsletters; and the format of eNewsletters according to preferences. The row labelled “Meet all 6 criteria” consists of a binary variable, calculated where “1” indicates that all needs of the respondents were being met in these categories and “0” if otherwise. In the last row labelled “Average score”, a score out of 6 for each consumer group was calculated. These results clearly show that the scores for 2010 and 2013 are similar for the different consumer groups. These results suggest that the CHI theoretical framework guiding the identification of those elements needing to address different consumer group information needs, proved effective over time.

Furthermore, a logistic regression model was fitted to the data with the binary variable labeled “Meet all 6 criteria”, the year and consumer group as main effects. The year 2010 and Health Profession consumer group served as reference groups. Table 4 shows the key finding of an odds ratio of 1.18 (95% CI: 0.67, 2.09), suggesting that information needs of respondents were equally met in 2013 as compared to 2010, as adjusted for consumer groups.

**Table 3:** 2010 and 2013 by consumer groups and 6 criteria on meeting information needs.

	2010					
**% Yes responses**	**Health Profession (n = 22)**	**Public (n = 34)**	**Policy (n = 6)**	**Education (n = 49)**	**Science (n = 20)**	**Average 2010**
**Meet Needs**	95.5	100.0	66.7	93.9	90.0	93.9
**Reliable**	95.5	100.0	83.3	98.0	100.0	97.7
**Use Info**	63.6	88.2	50.0	65.3	70.0	71.0
**Read Articles**	86.4	91.2	100.0	91.8	80.0	89.3
**eNewsletter useful**	100.0	97.1	83.3	95.9	100.0	96.9
**eNewsletter format**	90.9	91.2	66.7	91.8	90.0	90.1
**Meet all 6 criteria**	**54.5**	**73.5**	**33.3**	**53.1**	**50.0**	**57.3**
**Average score**	**88.6**	**94.6**	**75.0**	**89.5**	**88.3**	**89.8**
	**2013**					
**% Yes responses**	**Health profession (n = 24)**	**Public (n = 15)**	**Policy (n = 5)**	**Education (n = 24)**	**Science (n = 15)**	**Average 2013**
**Meet Needs**	100.0	95.8	100.0	87.5	93.3	94.0
**Reliable**	100.0	91.7	100.0	100.0	86.7	95.2
**Use Info**	80.0	62.5	100.0	75.0	60.0	71.1
**Read Articles**	93.3	79.2	80.0	100.0	93.3	90.4
**eNewsletter useful**	100.0	95.8	100.0	95.8	93.3	96.4
**eNewsletter format**	100.0	91.7	100.0	95.8	93.3	95.2
**Meet all 6 criteria**	**73.3**	**54.2**	**80.0**	**62.5**	**53.3**	**61.4**
**Average score**	**95.6**	**86.1**	**96.7**	**92.4**	**86.7**	**90.4**

**Table 4:** Logistic regression model using 2010 and Health Profession as reference group.

Meet All 6 Criteria	Odds Ratio	*p*-Value	95% Confidence Interval	
*2010*	*1.000*			
2013	1.184	0.559	0.672	2.087
*Health profession*	*1.000*			
Public	1.266	0.568	0.563	2.850
Education	1.463	0.296	0.716	2.987
Policy	0.917	0.894	0.256	3.287
Science	0.812	0.615	0.361	1.828

A final open-ended question, again not mandatory, enabled respondents to give general overall comments as to how they perceived information needs as being met. Twenty-four of the 83 respondents used this opportunity with 1 in the Public group, 6 in Education, 1 in Policy, 9 in Health Profession and 8 in the Science consumer groups. Qualitative data analysis led to the emergence of 5 main themes. *Meeting information needs* (48%, n = 12), many comments were expressed on the appreciation and usefulness of AfroAIDSinfo as being an important resource on HIV/AIDS prevention. Specific comments were made on *Usefulness of articles* (16%, n = 4), perceived as informative and scientifically sound, and thus enabling respondents to improve their knowledge and enabling them to educate others. *Meeting user preferences* (16%, n = 4), included comments of relevancy, reusability and suitability of format for educative purposes. *Reliability of content* (12%, n = 3) included referencing in articles that enabled validating and trustworthiness of content. *Usefulness of the eNewsletter* (8%, n = 2) was seen as very informative and useful for further dissemination. These results clearly supported the quantitative results as in Table 3.

## 4. Discussion

Establishing if the AfroAIDSinfo consumer information needs were met was the main purpose of this study. Thus, the findings have been interpreted according to the key areas contained in the theoretical framework.

### 4.1. Analyzing Health Consumers’ Preferences, Information Needs and Use

Eysenbach’s landmark paper on CHI [9], emphasizes the importance of knowing who your health consumers are, as this tends to inform consumers’ health decisions. 82% of respondents in this study were from sub-Saharan African countries, with the participants’ demographic profiles showing that the highest response rates were amongst university post-graduates, females and those from the 35–54 age group. These findings are similar to those in a review on health-seeking behavior conducted in Europe [16]. Powell *et al.* [17] further corroborated the findings of this study by referring to the increasing evidence that this particular profile has been associated with the most frequent health-seeking behavior on the Internet. 

This finding implies health consumers fitting this profile are the most active and should thus be accommodated in health information systems and health Web sites. However, methods for equitable access to health information should be researched to benefit other age groups as well. 

Of the five targeted consumer groups, the highest response rates were from health professionals and educators who became members to conduct research on HIV/AIDS prevention and to provide health care services, respectively. They also highly valued reliability and usability of the AfroAIDSinfo Web portal. When qualitatively examining how information on the Web portal was reused, the main themes which emerged were “*Education*”, “*Referencing and Research*” and “*Dissemination*”. This finding closely relates to Woolf’s T2 description of “Translational Research” [18] which is the field of study seeking to close the gap between knowledge generated from clinical research and the tailoring of that knowledge for optimal understanding by patients. This goes beyond the idea of “from bench to bedside” and places the emphasis on the implementation of communication methods to ensure that information reaches the intended end users in a comprehensible format. Woolf continued to emphasize how more patients might benefit from a healthcare system that spends more resources in researching *how* current treatments can be effectively administered which for instance, implies to be found acceptable by patients, in contrast to researching ever-new treatments that cannot be effectively utilized [18].

The increased availability of ICTs and the Internet created opportunities for people from other professions and community members other than health professionals to access health information. Notably, 87% of participants in this study confirmed that their professions were related to the consumer groups in which they were registered, implying that information on HIV/AIDS was critically sought across a wide spectrum of people responding to this epidemic. These included patients and community members but also health professionals, educators, scientists and policy makers alike. For example, the study found that the three main reasons for membership was to obtain reliable HIV/AIDS information for various educational purposes, followed by the conduction of AIDS research and the provision of health care services (Figure 1). In this context Weaver *et al.* [19] argues that consumers who are ill or healthy are motivated for seeking health or wellness information. Clearly, the identification and segmentation of consumer groups in the AfroAIDSinfo Web portal as suggested by the theoretical framework of CHI and the WPKTM contributed to the tailoring of HIV/AIDS information for those who are ill or responsible for policy formulation, conducting research, as well as caring or educating others. 

### 4.2. Developing Approaches to Integrate Consumer Preferences in Information Systems

CHI suggests that once a consumer group has been identified, attempts should also be made to integrate information needs and preferences of consumers in information systems. This led to the development of the WPKTM that ensures that health information is relevant and needed, classified according to health literacy levels and highlights relevance of new knowledge, while avoiding information overload and encouraging utilization of this knowledge. This concept is strongly in line with the review performed by Revere *et al.* [20] who identified major barriers to information access as reliability of resources, the credibility of information, overload of information as well as a lack of time to find information, indicating a need to have readily extrapolated information, which is presented according to specific needs. 

By combining the key areas of CHI and the elements of the WPKTM, it was possible to develop a health resource providing relevant HIV/AIDS information to diverse groups. This theoretical framework also places emphasis on monitoring and evaluation to continuously meet consumers’ information needs. Conducting regular eSurveys reiterates the high satisfaction rates of respondents in this study. Alpay *et al.* [21] who investigated improvement methods for consumers to understanding of health information, similarly suggested through guidance of a theoretical framework. 

The fact that participants indicated their use of information garnered from the AfroAIDSinfo Web portal for referencing, research, education and further dissemination indicated the content was found to be not only reliable and up to date, but also indicates its ease of understandability. This led to three of the five consumer groups reporting their information needs were being fully met 100% with the other two groups showing remarkably high scores at 93% and 88% respectively. 

### 4.3. Research Influence of CHI Outcomes on Public Health

For this study, the theoretical framework confirmed the meeting of information needs, the quality and reliability of the information, the usefulness of this information and the tailoring of information according to user preferences. Collectively, these factors should enable all consumer groups to be able to make informed choices on disease prevention, improvement in health-seeking behavior as well as promotion of health—according to the definition of public health [22] as well as the ultimate aim of CHI [9]*.* The consumer groups in this study rated the meeting of their collective needs with high scores which were also corroborated qualitatively. 

Of the critical needs expressed in literature for improvement of public health outcomes include accessibility of information, tailoring of information according to user needs while uncertainty whether information is reliable and overload of information were seen as barriers to public health [20]. In this context, changes that were made to the way information was communicated to consumers since the previous eSurvey in 2010 have been received positively with 95% of respondents indicating that they found these additions useful. The comparison between the two eSurveys has also indicated that the CHI theoretical framework applied in conjunction with the WPKTM has enabled not only the tailoring of content, but also the deliverance of information to specific consumer groups has been well received and constant. This is an indication that the methods applied have proven to be useful over an extended period of time, not just in the short term or as a once-off intervention, making a valuable public health contribution on decision-making to diverse groups.

Health benefits were reinforced through the themes which emerged in open-ended questions. For instance, respondents often referred to the educational value of the AfroAIDSinfo resources that brought about personal empowerment to make informed decisions as well as to educate people in their areas of work. Consequently, Eysenbach [6] described consumer education and empowerment as contributions eHealth interventions should make to improve public health outcomes. Recognizing the potential of eHealth, the South African Department of Health emphasized its aim to establish eHealth as an integral part of the transformation and improvement of healthcare services in its new eHealth strategy [23].

In contrast, a review by McGray [24] emphasized the public health dilemma introduced by the vast amount of health information available on the Internet but the lack of health literacy, defined as the ability to understand and act on information, by many consumers to make health-improving decisions. 

## 5. Conclusions

The need for up to date information, which is tailored and collated from reliable sources, is the predominant need in the study by Revere *et al.* [20]. Using the WPKTM as a foundation for identifying the needs of consumers, the AfroAIDSinfo Web portal has collated and tailored information for its five consumer groups. Determining the demographics of these different groups has allowed for content as well as modes of delivery to be modified according to the needs of each consumer group. This has led to a high degree of satisfaction with not only the information on AfroAIDSinfo, but also the way in which it was presented to consumers.

A high level of confidence in the information as well as the finding of it to be a reliable source, which met respondent preferences, was also expressed. There was strong evidence in the comparison between the eSurvey in 2010 and the latest in 2013 that information needs were being met equally.

The WPKTM approach is not limited to the field of HIV/AIDS, and could be used in any topic of public health to improve the translation and dissemination of information to the end user. A further improvement to the WPKTM might be an increased focus on direct, personal interaction between consumers on the AfroAIDSinfo Web portal and experts who supply the content as shown in the Revere *et al.* [20] study. 

Furthermore, the study purpose was not to sample, but rather to include the whole AfroAIDSinfo population. Therefore, a major study limitation was the low overall response rate (4.6%). Participation in previous AfroAIDSinfo CHI studies might have created the perception among participants that their information needs are being met on a continuous basis, which discouraged participation. Then again, it is also well known that online survey response rates are low [25,26,27]. While incentives can increase response rates, it was not introduced in this study [26]. Rather, respondents were informed that their participation contributed to maintain a highly credible HIV/AIDS online resource. Eysenbach [27] explained that response rates could be as low as 1% for online surveys. Therefore, he suggested increasing external validity by, for instance, including questions that will enable comparison with historical data. For that reason, results from this study was compared to previous AfroAIDSinfo CHI studies, which showed that information needs in 2010 and 2013 were met equally. 

Further research should investigate and explore methods for equitable access to online health information for people outside the popular profile. This will also provide further evidence for up-scaling of the theoretical framework in other public health interventions. 

This study provided valuable knowledge to eHealth and CHI research from South and southern Africa. It has shown an effective approach to collect, collate and translate HIV/AIDS information with specific consumer groups in mind. The approach was deemed effective as the majority of the consumer groups expressed satisfaction with both the content of the AfroAIDSinfo Web portal and its modes of delivery. While recognizing the presence of scientific challenges, this study makes a very useful contribution in the area of eHealth and public health informatics where the current knowledge base is small.
